# Depicting External Crown Resorption in Syndromic Amelogenesis Imperfecta Associated With Junctional Epidermolysis Bullosa

**DOI:** 10.1002/cre2.70500

**Published:** 2026-10-29

**Authors:** Susanne Krämer, Sebastián Véliz, Anna‐Lena Hillebrecht, Ana Ortega‐Pinto, Colomba Besa, Ignacia Fuentes, Reinhard Schilke, Jens P. Bankstahl, Elias Walter, Wiebke Semper‐Hogg, Cristina Has

**Affiliations:** ^1^ Department of Dermatology, Medical Faculty and Medical Centre University of Freiburg Freiburg Germany; ^2^ Faculty of Dentistry University of Chile Santiago Chile; ^3^ Department of Periodontology, Dental Clinic Sigmund Freud University Vienna Austria; ^4^ Department of Prosthetic Dentistry, Center for Dental Medicine, Faculty of Medicine, Medical Center University of Freiburg Freiburg Germany; ^5^ DEBRA Chile Santiago Chile; ^6^ Centro de Genética y Genómica, Facultad de Medicina Clínica Alemana Universidad del Desarrollo Santiago Chile; ^7^ Facultad de Ciencias Biológicas Pontificia Universidad Católica de Chile Santiago Chile; ^8^ Department of Conservative Dentistry Periodontology and Preventive Dentistry, Hannover Medical School Hannover Germany; ^9^ Department of Nuclear Medicine Hannover Medical School Hannover Germany; ^10^ Department of Conservative Dentistry, Periodontology and Digital Dentistry LMU Hospital, LMU Munich Germany; ^11^ Department of Oral and Maxillofacial Surgery, Center for Dental Medicine, Faculty of Medicine, Medical Center University of Freiburg Freiburg Germany

**Keywords:** dental morphology, developmental biology, enamel, genetics, mucocutaneous disorders, osteoclasts

## Abstract

**Objectives:**

To characterize External Crown Resorption (ECR) in patients with Syndromic Amelogenesis Imperfecta (SAI) associated with junctional epidermolysis bullosa (JEB).

**Materials and Methods:**

A multicentre retrospective study was conducted at two centers (Germany and Chile). Analyzed variables included dentition, tooth, surface, age, severity, progression, treatment strategy, and clinical outcome.

**Results:**

One hundred and seventy teeth affected with ECR (60 primary, 110 permanent) from 30 patients were included. Patients presented an average of 5.7 ± 4.0 affected teeth. Within this cohort, in primary dentition, incisors represented 88% of affected teeth, 8% canines, and 3% molars. In permanent dentition, 62% were incisors, 19% molars, 10% canines, and 9% premolars. Eight teeth showed two independent initiation sites, giving 178 affected surfaces in 170 teeth; 85.4% of these surfaces were incisal/occlusal, 12.9% proximal, and 1.7% vestibular/palatal. Age of detection varied from 8 months to 8.7 years (primary dentition) and from 5.2 to 22 years in permanent dentition. Severity analysis revealed that posterior teeth presented a higher proportion of severely compromised molars (61.9%), while anterior teeth presented a higher proportion of more moderate (57.4% permanent) and mild (37.7% primary) compromise. Observed progression intervals, based on 39 teeth, ranged from 4 months to 9.3 years. Imaging studies, treatment strategies, and their outcomes are presented. Restoration was associated with a lower frequency of further structural loss than preventive strategies alone. A provisional radiographic screening protocol is proposed.

**Conclusion:**

ECR can affect primary and permanent dentition, leading to tooth loss if left unrestored. The radiographic screening protocol for early diagnosis and treatment of ECR proposed in this article can aid in preserving teeth.

## Key Findings

- ECR can occur in primary dentition, as early as 8 months of age.
- ECR most commonly starts from the incisal edge/occlusal cusp, but can also start from the proximal, vestibular, and palatal/lingual aspects of the tooth.
- In most cases, a thin layer of dentine covers the pulp. The pulp tissue remains vital.
- On average, 5.7 teeth were affected per patient. Incisors represented the largest proportion of affected teeth in this cohort.
- Semilunar edges of incisors can be a result of external crown resorption in AI/SAI.
- Resorption of primary incisors can be diagnosed as early as 8 months; if unerupted at the age of 12 months, PAR should be considered as an ECR screening tool.
- Resorption of permanent incisors can be diagnosed from the age of 5.2 years. Screening of ECR should be considered in unerupted incisors from the age of ~6.
- Resorption of permanent molars can be diagnosed from the age of 7.4 years. Screening of ECR should be considered in unerupted first molars with OPG from the age of ~7.
- Resorption can be diagnosed from the age of 8.7 years in premolars, 9.3 years in canines, and 11 years in second molars. Screening should consider OPGs from the age of ~10 until full eruption.

## 1 Introduction

Amelogenesis Imperfecta (AI) is a heterogeneous genetic condition affecting the enamel formation, structure, and clinical appearance of the entire dentition with hypoplastic, hypomature, hypocalcified, or combined types. AI can be inherited as an isolated form or as a manifestation of a syndrome, in which case it is termed Syndromic Amelogenesis Imperfecta (SAI) (Crawford et al. 2007). More than 70 genes are known to be involved in its pathogenesis. Non‐syndromic AI is linked with genes encoding enamel matrix proteins such as amelogenin (*AMELX*), enamelin (*ENAM*), and ameloblastin (*AMBN*); while SAI can be caused by mutations in a wide variety of genes, such as *FAM20A* in Enamel‐renal syndrome, *CNNM4* in Cone‐rod dystrophy, and *LAMA3, LAMB3, LAMC2,* and *COL17A1* resulting in Junctional Epidermolysis Bullosa (JEB) (Bloch‐Zupan et al. 2023).

In addition to structural enamel abnormalities, individuals with AI/SAI can present with other dental anomalies, including external crown resorption (ECR), delayed tooth eruption, pulp calcification, and congenitally missing teeth (Collins et al. 1999).

Odontoclast‐mediated ECR of AI‐affected teeth during pre‐eruptive and peri‐eruptive periods was first described by Weinmann in 1945 as a resorption starting at the tip of the crown, in that part of the crown adjacent to the oral epithelium. Odontoclasts resorb enamel and dentine, forming resorption lacunae filled with connective tissue; the pulp chamber remains protected by a thin wall of predentine and is only rarely opened. Bone formation can occur in the connective tissue invading the tooth. If this occurs, the newly formed bone has no connection with the alveolar bone (Weinmann et al. 1945). The process is asymptomatic, fast, aggressive, and extensive. In most patients, it is identified radiographically before teeth emerge into the oral cavity. It does not affect the root (Bhatia et al. 2015; Koruyucu et al. 2018).

ECR has been described in unerupted teeth in all three types of isolated AI (hypocalcified, hypoplastic, and hypomature) (Collins et al. 1999). In SAI, it has been described in patients with Enamel‐Renal‐Syndrome (MacGibbon 1972; Phakey et al. 1995; Wang et al. 2013), and JEB (Urzúa et al. 2021).

Available evidence shows that ECR occurs in permanent dentition, and the most frequently affected teeth are premolars and molars (Morrier et al. 2008), with few canines affected (Weinmann et al. 1945). The number of teeth affected per patient is highly variable: from isolated teeth (one to three per patient) (Phakey et al. 1995; Morrier et al. 2008; Alvarez et al. 2022), to multiple teeth affected (four to six) (Weinmann et al. 1945; Korbmacher et al. 2007), up to severe compromise (more than 12) (Miloglu et al. 2011). Conservative treatments are possible, and the prognosis is favorable if lesions are treated early (O'Neal et al. 1997). However, when the diagnosis is late, extractions are often performed (Korbmacher et al. 2007).

Early diagnosis and treatment are key to preventing pulp involvement (Davidovich et al. 2005). Understanding the epidemiological data on age of detection, type of teeth, and dentition affected will allow the establishment of screening protocols for early interventions to avoid tooth loss and improve the quality of care for patients with AI (Okutan et al. 2025).

The aim of this study is to characterize ECR in patients with SAI associated with JEB: epidemiological data, progression, treatment strategies, and outcomes, as a basis for an early radiographic screening protocol and recommendations on key therapeutic strategies for tooth‐preserving management.

## 2 Materials and Methods

### 2.1 Study Design, Settings, and Participants

Multicentre retrospective longitudinal cohort study analyzing medical records, genetic diagnosis, clinical images, dental radiographs, and histological samples at two clinical centers: Dermatology Department, University Clinic Freiburg, Germany (UKF), and Special Care Dentistry Clinic, University of Chile (UCH). Inclusion criteria: confirmed diagnosis of JEB (Has et al. 2020) and ECR. Exclusion criteria were a significant loss of tooth structure, preventing the distinction between ECR and caries. Eighteen patients from the Chilean center were included in a previous single‐center prevalence study of crown resorption (Besa‐Witto et al. 2025). That study reported prevalence only; the present study adds the German center, longitudinal progression, histopathology, treatment strategies, and outcomes. No analysis is duplicated. Ethical approval was granted by the Institutional Review Boards of the Albert‐Ludwigs‐Universität, University Medical Center Freiburg, Germany (EK Freiburg 21‐1171), and the North Metropolitan Health Service, Chile (002/2023). A convenience sample was established, including all patients meeting the inclusion criteria at UKF from January 2003 to December 2025 and at UCH from January 2005 to December 2025. The results were reported following the STROBE Statement for cohort studies (von Elm et al. 2007).

### 2.2 Variables and Data Source

The variables analyzed included AI (Crawford et al. 2007), JEB subtype (Has et al. 2020), dentition, tooth, surface, age at detection, severity, progression, treatment strategy, and clinical outcome. ECR severity was categorized as: mild resorption—involves up to the outer third of the dentine; moderate resorption—involves between one‐third and two‐thirds of the dentine; severe resorption—affects more than two‐thirds of the dentine with a thin layer of dentine remaining visible; and complete resorption—discontinuity of the dentine layer resulting in a communication between resorptive and pulp tissue or pulp exposure. Periapical lesions were included in this category. Severity is reported as the maximum severity recorded during follow‐up. Observed progression intervals were analyzed in teeth with information from multiple visits; because examination intervals were not standardized, these intervals are bounded by the timing of available examinations. Age of detection denotes the age at which resorption was first identified and does not represent the age of onset. Treatment strategies were grouped into: preventive/no treatment, gingivectomy, direct restorations (composites, amalgams, and glass‐ionomer‐cement), interim crowns (stainless steel or polymethyl methacrylate), ceramic crowns, endodontic therapies, extractions, and dental implants. These categories were not mutually exclusive: a tooth could receive sequential or combined interventions. Co‐occurrence is reported in Supporting Information S1: Table S1. Percentages of treatment strategies are expressed over the number of tooth treatments per severity group rather than the number of teeth, as a tooth could receive more than one strategy; gingivectomy is reported over the same denominator but excluded from it, as it is a gingival and not a dental procedure. Missing data included 32 teeth (18.8%) that had no treatment recorded (15 mild, 9 moderate, and 8 severe) and were excluded from these denominators. Outcomes were classified into: complete tooth structure—natural or restored teeth that maintained or recovered their structure; structural loss—loss of enamel/dentine, complete tooth loss. Outcome was assessed at the last available clinical or radiographic examination; teeth without a subsequent examination were recorded as not assessable.

### 2.3 Image Assessment/Examiner Calibration

Diagnosis of ECR was established based on dental radiographs of unerupted teeth and clinical images of erupted teeth, as well as available clinical records. In unerupted and partially erupted teeth, the diagnostic criteria were irregular, moth‐eaten radiolucencies affecting the coronal dentine, with no evidence of reactionary or reparative dentine. In erupted teeth, ECR was distinguished from developmental enamel defects, attrition, post‐eruptive breakdown, and caries by the shape of the lesion (concavities or semilunar shapes in ECR, compared to smooth, flat worn‐off surfaces in attrition, fractured margins in post‐eruptive breakdown, and cavitation pattern of caries), the color (normal dentine color in ECR compared to chalky white, yellowish, light brown or dark brown dental tissues in caries, and/or developmental enamel defects); and, when available on the clinical records, the hardness of the dentine (normal hardness in ECR compared to soft dentine in caries). Where serial images were available, an initial straight incisal edge acquiring a semilunar contour confirmed a resorptive process rather than a developmental origin.

To reduce the risk of bias, clinical and radiographic images were independently assessed by two calibrated clinicians (Interexaminer Cohen's Kappa 0.934, Intraexaminer Cohen's Kappa SK: 1.0, SV 0.92). Disagreements were resolved by a third reviewer (C.B.). Training and calibration on developmental enamel defects were based on diagnostics by Dabiri (Dabiri et al. 2018).

### 2.4 Histopathological Procedure

Eight samples from patients with *LAMB3* genetic variants were evaluated: five teeth (three molars, one premolar—patient 16, Figure 1E; and one trephined lateral incisor—patient 6, Figure 2D) and three gingival tissues (excised from resorptive cavities during gingivectomies: patient 4, Figure 1A and patient 19, Figure 1F). The samples were fixed in 10% buffered formalin; the teeth were decalcified in EDTA for 6–10 weeks. All samples were processed by routine histopathological technique and stained with haematoxylin‐eosin. The sample from patient 15 was previously published (Urzúa et al. 2021).

**Figure 1:**
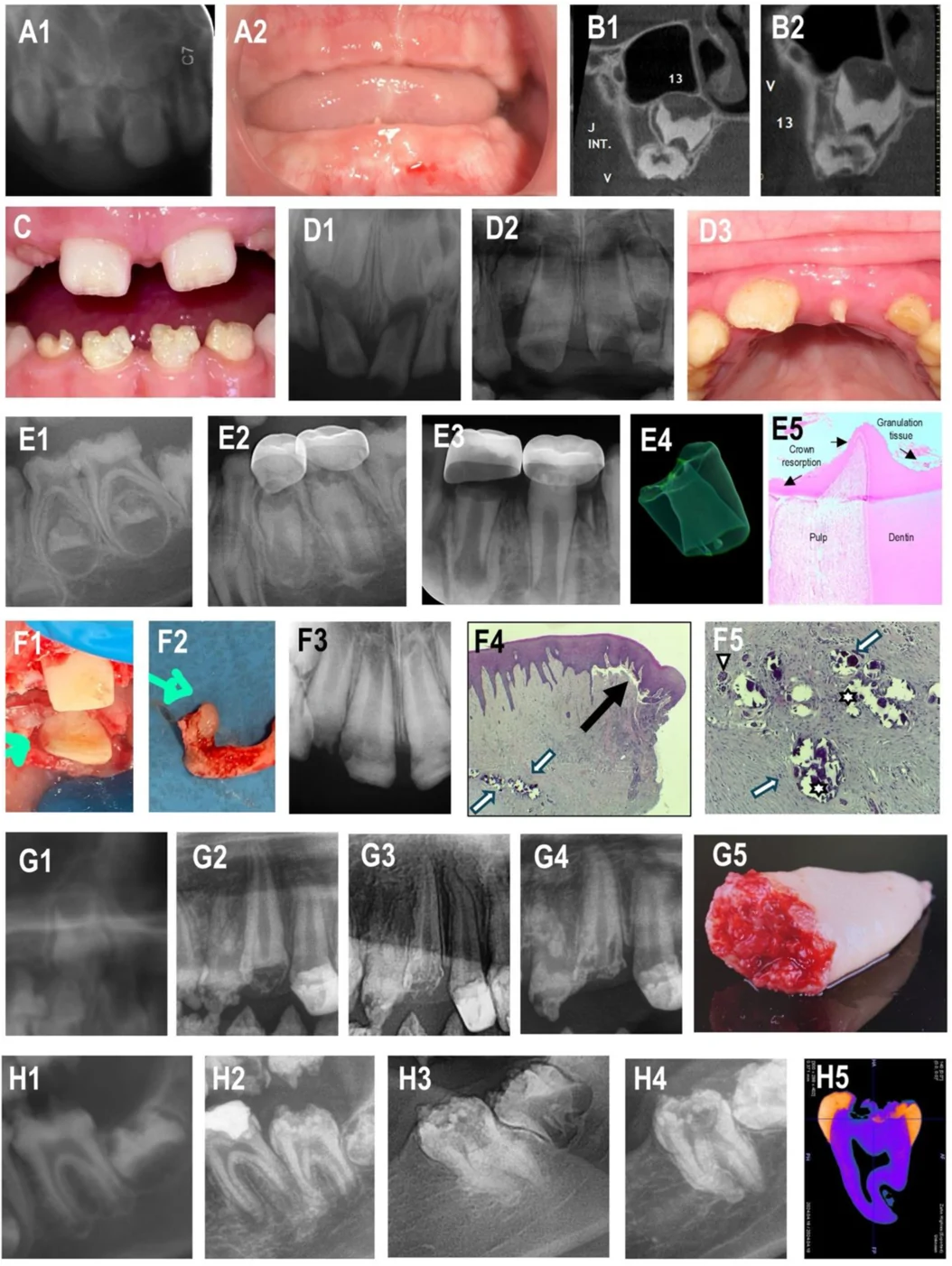
Clinical examples of external crown resorption and its progression. External crown resorption in Amelogenesis Imperfecta associated with junctional epidermolysis bullosa. (A) Severe crown resorption of unerupted primary upper right central incisor in a 10‐month‐old patient. A1: radiograph, A2: clinical image. (B) Resorption of retained upper left second primary molar in CBCT study. B1: crown integrity at the age of 7 years 6 months, B2: resorption of the palatal cusp becomes evident at the age of 8 years 8 months. (C) Mild ECR of lower incisors #32, #31, and #41; and moderate ECR of #42 at the age of 7 years 8 months. (D) Severe ECR of permanent upper left central incisor (#21). D1: radiograph at the age of 3 years 11 months. The incisal edge of both upper central incisors can be observed, both with straight edges. D2: Radiograph at the age of 6 years 7 months. Severe resorption of peri‐eruptive #21 can be observed, with a “semilunar edge.” D3: Clinical picture at the same age. Only the mesial tip has erupted. (E) Complete ECR of a lower left first premolar. E1: 4 years 7 months crowns of #34 and #35 during the mineralization process. E2: 9 years 6 months: #34 and #35 during root formation. E3: 9 years 10 months only 4 months later, complete crown resorption of unerupted #34 is detected. E4: Micro‐CT image of extracted #34. E5: Histology of the same tooth. Extensive coronal resorption, granulation tissue next to the resorbed area. Vital pulp without inflammation. Hematoxylin‐eosin stain, original magnification ×25. (F) Moderate resorption starting from the distal aspect of the upper right central incisor diagnosed at the age of 7 years 10 months. F1: Picture taken during gingivectomy, right after the gingival tissue was removed from the resorptive lacunae. The lesion affects the distal and palatal aspect of the tooth. Color and hardness of the dentine are not affected. F2: Removed gingival tissue. The arrow points at the area of the tissue that was located in the resorptive lacunae. The area adjacent to the resorptive lesion is glossy. F3: Radiograph of the same tooth. F4: Histology of the removed gingival tissue: subepithelial vesicle (black arrow) and gingival hamartoma/calcifications within the gingival tissue (white arrows). Magnification ×40. F5: Calcifications (white stars) and odontogenic epithelium (white arrows), magnification ×100. (G) Complete ECR in unerupted upper right canine #13. G1: 6 years 9 months: crown of #13 is completely formed. G2: At the age of 11 years 8 months, moderate ECR starting from the incisal edge can be observed. G3: 14 years 8 months: ECR has progressed to severe compromise; canine still unerupted. G4: 19 years 3 months: canine is still unerupted; resorption has progressed into the radicular dentine. G5: image of extracted canine at the age of 21 years. (H) ECR of unerupted permanent lower left second molar: H1: age 6 years 9 months #37 during the crown mineralization process, H2: age 11 years 8 months #37 during root development, unerupted, H3: age 14 years 8 months #37 still unerupted with clear signs of crown resorption, H4: age 19 years 3 months #37 and #38 still unerupted. Resorption has progressed. At the age of 21, the retained #37 and #38 were extracted. H5: Micro‐CT image of extracted #37.

**Figure 2:**
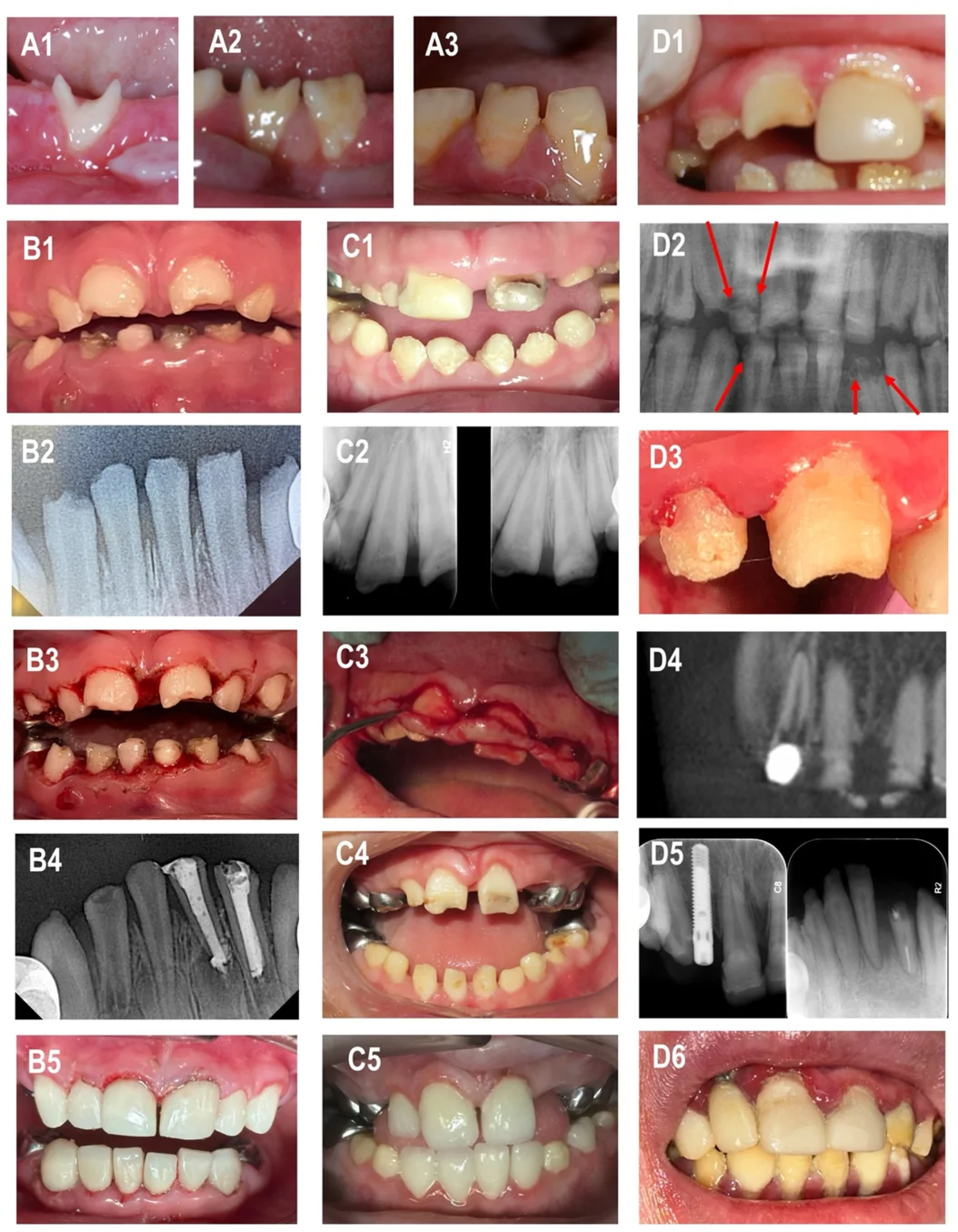
Treatment strategies for external crown resorption. Treatment strategies for ECR in SAI. (A) Progressive attrition of the lower incisor with moderate ECR, as no restorative treatment was provided. A1: 7.9 years, A2: 10 years, A3: 17 years. (B) Gingivectomy, pulpectomy, and interim crowns on primary dentition at 1.7 years. (C) Therapeutic approach in permanent dentition: C1: direct restorations of upper incisors in the eruption process. C2: Periapical radiographs; resorption (semilunar edges) can be recognized on both central incisors. C3: gingivectomy; C4: healed gingival tissue after surgery. Semilunar edges are clearly recognizable. C5: interim crowns on permanent incisors. (D) Severe resorption with radicular dentine compromise and implant. D1: lateral incisor (12) during the eruption process. No defect is visible (#11 presents moderate ECR at the incisal edge; #21 is restored with an interim crown). D2: On OPG, ECR on the proximal surfaces of #12, #32, and #42 was diagnosed (red arrows). D3: clinical picture of #12. Note gingival tissue in the disto‐cervical area, where resorption is taking place. D4: an attempt to stop resorption was performed; however, the condition progressed into the radicular dentine. D5: the lateral incisor had to be extracted and replaced by an implant. Lower lateral incisor #42 was treated with endodontics. D6: ceramic crowns on #12 and #42, interim crowns on the other incisors. Gingival inflammation is present due to difficulties in maintaining adequate oral hygiene.

### 2.5 Statistical Methods

Descriptive statistical analysis was performed for all variables. Categorical variables are expressed as numbers and percentages; numerical variables are expressed as means, standard deviations, or medians and ranges. The distribution of quantitative variables was assessed using visual inspection of histograms. Spearman's rank correlation coefficient was used to analyze the association between specific variables (N° of visits/N° of teeth with ECR). Severity was first modeled as an ordinal outcome (mild < moderate < severe < complete). The proportional‐odds assumption was formally tested and was not met (likelihood ratio test for nominal effects, *p* = 0.003), due to the absence of mildly affected premolars. Severity was therefore dichotomized (severe or complete vs. mild or moderate) and analyzed by generalized estimating equations with an exchangeable working correlation and robust standard errors, with permanent incisors as the reference group. The ordinal model with cluster‐robust standard errors at a patient‐level bootstrap (2000 resamples) is reported as a sensitivity analysis. Odds ratios are presented with 95% confidence intervals. The affected tooth surface was analyzed with Fisher's exact test with Monte‐Carlo simulation, using the affected surface rather than the tooth as the unit of analysis (178 surfaces in 170 teeth). Statistical significance was considered with a *p*‐value < 0.05 (two‐sided). Because teeth within a patient are not independent observations, all inferential analyses account for clustering at the patient level. A mixed‐effects ordinal model with a random intercept for patient was also fitted; the between‐patient variance was negligible (intraclass correlation < 0.001) and the working correlation estimated by the generalized estimating equations was likewise close to zero (*α* = −0.052, SE = 0.057). With 24 patients contributing permanent teeth, this indicates that clustering could not be detected, rather than that it is absent, and all inferential results are therefore reported as exploratory. Analyses were performed on complete cases with no imputation. The RStudio 2025.05.0 software was used.

## 3 Results

### 3.1 Sample Description

The study group consisted of 30 patients with SAI and ECR associated with JEB (UKF *n* = 11, UCH *n* = 19). All exhibited generalized hypoplastic AI: severe generalized *n* = 28, generalized pitting *n* = 2. JEB subtypes were: severe (n = 10) and intermediate (*n* = 20). Genetic diagnoses were available in 28 patients: 27 with pathogenic variants in genes encoding laminin 332: *LAMA3* (*n* = 2), *LAMB3* (*n* = 24), *LAMC2* (*n* = 1), and 1 with *COL17A1*. Sex distribution was 12 males and 18 females. ECR diagnoses were based on 340 clinical photographs, 65 orthopantomographies (OPGs), 91 periapical radiographs (PAR), 13 cone‐beam computed tomography (CBCT) scans, 8 histological samples, and 2 Micro‐CT (Table 1).

**Table 1:** Description of participants.

Pt	Age 1st	Age last	Sex	Center	Gene	Variant (cDNA)	Protein	JEB	AI	Teeth	Visits	Photos	OPG	PAR	CBCT	Others
1	7.6	20.3	M	UKF	*LAMA3*	NA	R943*; R1159*	intermediate	severe	5	11	8	5	0	0	0
2	6.8	21.3	F	UKF	*LAMA3*	c.2008+1G > T	SS	intermediate	pitting	7	6	13	5	2	0	1 M
3†	0.7	3.8	F	UCH	*LAMB3*	c.3228+1G > A	SS	severe	severe	4	6	12	2	0	0	0
4	0.8	4.2	M	UCH	*LAMB3*	c.3228+1G > A	SS	severe	severe	4	3	4	1	7	0	1 H (G)
5	0.8	13	M	UCH	*LAMB3*	c.3228+1G > A	SS	intermediate	severe	15	10	24	4	3	4	0
6	0.9	16.7	F	UCH	*LAMB3*	c.3228+1G > A	SS	severe	severe	12	13	41	3	2	2	2 H (T, G)
7†	0.9	0.9	F	UCH	*LAMB3*	c.282G > A; c.3228+1G > A	W94*; SS	severe	severe	2	1	9	0	0	0	0
8	1.6	16.3	M	UCH	*LAMB3*	c.3228+1G > A	SS	intermediate	severe	13	14	60	5	31	1	0
9†	1.7	1.7	F	UCH	*LAMB3*	c.2975delT; c.3228+1G > A	V992Gfs*38; SS	severe	severe	4	1	2	0	0	0	0
10†	1.7	1.9	F	UCH	*LAMB3*	c.3228+1G > A	SS	severe	severe	10	3	4	0	4	0	0
11†	1.7	2.1	F	UCH	*LAMB3*	c.3228+1G > A	SS	severe	severe	8	3	3	0	8	0	0
12	1.8	19.9	M	UCH	*LAMB3*	c.3228+1G > A	SS	intermediate	severe	3	15	43	8	4	2	0
13†	2.1	5.8	M	UCH	*LAMB3*	c.3228+1G > A	SS	severe	severe	3	2	4	1	0	0	0
14	2.2	5.8	F	UCH	*LAMB3*	c.3228+1G > A	SS	intermediate	severe	10	6	5	2	8	0	0
15†	3	10	F	UCH	*LAMB3*	c.957_958dup77; c.3228+1G > A	N345Kfs*77; SS	severe	severe	10	7	12	3	6	0	1 H (T)
16	4.6	13.1	F	UCH	*LAMB3*	c.939C > A; c.973T > C	C313*;C325R	intermediate	severe	12	8	24	4	7	1	2 H (T), 1 M
17†	7	7	F	UCH	*LAMB3*	c.3228+1G > A; c.3268del5	SS;D1090Vfs*9	intermediate	severe	3	1	2	0	0	0	0
18	7.7	7.7	M	UKF	*LAMB3*	c.628G > A;c.1903C > T	E210K; R635*	intermediate	severe	4	1	3	1	0	0	0
19	7.9	11.7	F	UCH	*LAMB3*	c.3228+1G > A	SS	severe	severe	6	4	8	4	8	1	1 H (G)
20	8.6	11	F	UKF	*LAMB3*	c.628G > A; c.1903C > T	E210K; R635*	intermediate	severe	2	3	3	0	0	0	0
21	8.4	13.9	M	UKF	*LAMB3*	c.2701+1G > A	SS	intermediate	severe	2	4	2	4	0	0	0
22	10.6	18.6	F	UKF	*LAMB3*	c.2701+1G > A	SS	intermediate	severe	3	6	14	5	0	1	0
23	11	19.9	F	UKF	*COL17A1*	c.3686dupC	V1231Cfs*11	intermediate	pitting	3	2	0	1	0	0	0
24	11	13	F	UKF	NA	NA	NA	intermediate	severe	6	2	2	2	0	0	0
25	11.4	15.9	M	UCH	*LAMB3*	c.3228+1G > A	SS	intermediate	severe	8	6	6	3	1	1	1 H (T)
26	12.8	12.8	M	UKF	*LAMB3*	c.628G > A; c.1903C > T	E210K; R635*	intermediate	severe	1	5	7	0	0	0	0
27†	14.4	15.7	M	UCH	NA	NA	NA	intermediate	severe	6	3	10	1	0	0	0
28	16.2	32.7	M	UKF	*LAMB3*	NA	E210K; R635*	intermediate	severe	1	5	6	0	0	0	0
29†	22	26	F	UCH	*LAMB3*	c.3228+1G > A	SS	intermediate	severe	1	2	6	0	0	0	0
30	9.8	24	F	UKF	*LAMC2*	NA	R245*	intermediate	severe	2	1	3	1	0	0	0

*Note:* Ages 1st/last: age at the first and last dental examination.

Abbreviations: AI, amelogenesis imperfecta (severe, severe generalized hypoplastic; pitting, generalized pitting hypoplastic); CBCT, cone‐beam computed tomography; JEB, junctional epidermolysis bullosa; OPG, orthopantomography; PAR, periapical radiograph; UCH, University of Chile; UKF, University Medical Center Freiburg. Others, additional studies performed (H, histology; G, Gingival tissue; T, Tooth; M, micro‐computed tomography); NA, not available.

^†^ Deceased.

### 3.2 Epidemiological Data

A total of 170 teeth were diagnosed with ECR, 60 primary and 110 permanent teeth.

#### 3.2.1 Number of Teeth Per Patient

On average, patients had 5.7 teeth with ECR (range 1−15, SD ± 4.0). The average number of dental visits with a diagnosis of ECR was 5 (range 1–15). A significant correlation between the number of visits and teeth diagnosed was observed (*Spearman ρ* = 0.509, *p* = 0.004). This association most likely reflects the intensity of surveillance rather than a biological relationship, since patients examined more frequently had more opportunities for ECR to be detected (Table 1, Supporting Information S1: Figure 1).

#### 3.2.2 Dentition and Teeth With ECR

In primary dentition, incisors represented 88% of affected teeth in this cohort (*n* = 53). Five canines were affected, while only two molars were diagnosed. In permanent dentition, incisors accounted for 62% of the affected teeth (*n* = 68), followed by molars (19%, *n* = 21), canines (10%, *n* = 11), and premolars (9%, *n* = 10) (Supporting Information S1: Figure 2).

#### 3.2.3 Surface Affected

The resorption process started from the tip of the crown in 85.4% of the 178 affected surfaces (71.3% incisal edge, 14.0% occlusal aspect), while 12.9% started from proximal areas (mesial/distal) and 1.7% from vestibular or palatal/lingual surfaces. In eight teeth, it started from two surfaces independently (Table 2, Figure 1A,C,D,G incisal; E, H occlusal; F proximal). Significant differences were observed in the distribution of affected surfaces (Fisher's exact test with Monte‐Carlo simulation, 10,000 replicates; *p* < 0.001). Incisors and canines were mostly affected on incisal surfaces; premolars and molars predominantly on occlusal and proximal surfaces.

**Table 2:** External crown resorption in amelogenesis imperfecta associated with junctional epidermolysis bullosa: Age at detection, severity, and affected surface.

			Severity, *n* (%)				Affected surface, *n* (%)					
Dentition/tooth group	Teeth (*n*)	Age at detection, years (median, range)	Mild	Moderate	Severe	Complete	Incisal	Occlusal	Proximal	Vestibular	Palatal	Teeth with longitudinal data (*n*)
*Primary dentition*	60	1.7 (0.7–8.7)	25 (41.7)	12 (20.0)	16 (26.7)	7 (11.7)	58 (96.7)	1 (1.7)	1 (1.7)			1
Incisors	53	1.7 (0.7–4.9)	20 (37.7)	12 (22.6)	15 (28.3)	6 (11.3)	53 (100.0)					0
Upper incisors	21	1.7 (0.7–3)	6 (28.6)	6 (28.6)	7 (33.3)	2 (9.5)	21 (100.0)					0
Lower incisors	32	1.7 (0.7–4.9)	14 (43.8)	6 (18.8)	8 (25.0)	4 (12.5)	32 (100.0)					0
Canines	5	1.7 (1.7–2.2)	5 (100.0)				5 (100.0)					0
Molars	2	6.5 (4.2–8.7)			1 (50.0)	1 (50.0)		1 (50.0)	1 (50.0)			1
*Permanent dentition*	110	9.6 (5.2–22)	29 (26.4)	46 (41.8)	25 (22.7)	10 (9.1)	69 (58.5)	24 (20.3)	22 (18.6)	2 (1.7)	1 (0.8)	38
Incisors	68	8.4 (5.2–22)	21 (30.9)	39 (57.4)	4 (5.9)	4 (5.9)	59 (83.1)		10 (14.1)	2 (2.8)		19
Upper incisors	35	8.6 (5.6–22)	13 (37.1)	17 (48.6)	4 (11.4)	1 (2.9)	29 (78.4)		8 (21.6)			10
Lower incisors	33	7.9 (5.2–14.8)	8 (24.2)	22 (66.7)		3 (9.1)	30 (88.2)		2 (5.9)	2 (5.9)		9
Canines	11	11.4 (9.3–20)	5 (45.5)	4 (36.4)	1 (9.1)	1 (9.1)	10 (90.9)		1 (9.1)			4
Premolars	10	12.3 (8.7–17)		1 (10.0)	7 (70.0)	2 (20.0)		8 (80.0)	2 (20.0)			7
Molars	21	11.4 (7.4–20)	3 (14.3)	2 (9.5)	13 (61.9)	3 (14.3)		16 (61.5)	9 (34.6)		1 (3.8)	8
First molars	14	11 (7.4–14.8)	1 (7.1)	1 (7.1)	10 (71.4)	2 (14.3)		10 (55.6)	8 (44.4)			5
Second molars	4	13.1 (11–19.2)			3 (75.0)	1 (25.0)		4 (80.0)	1 (20.0)			2
Third molars	3	19.2 (13.4–20)	2 (66.7)	1 (33.3)				2 (66.7)			1 (33.3)	1
Total	170	7.9 (0.7–22)	54 (31.8)	58 (34.1)	41 (24.1)	17 (10.0)	127 (71.3)	25 (14.0)	23 (12.9)	2 (1.1)	1 (0.6)	39

*Note:* Severity is expressed as *n* (%) of the teeth in that row and refers to the maximum severity recorded during follow‐up. Affected surface is expressed as *n* (%) of the affected surfaces in that row (178 surfaces in 170 teeth), as eight teeth showed two independent initiation sites. Age at detection is the age at which resorption was first identified and does not represent the age of onset. Empty cells are zero.

#### 3.2.4 Age of Detection

In primary dentition, ECR was diagnosed as early as 8 months (Figure 1A shows ECR in a 10‐month old patient). Median age of detection for incisors was 1.7 years, for canines was 1.7 years, and for molars was 6.5 years (range 4.2–8.7 years). Interestingly, the molar with ECR at 8.7 years had a previous CBCT at 7.5 years, excluding resorption (Figure 1B).

In permanent dentition, median age of detection was incisors 8.4 years, first molars 11 years, canines 11.4 years, premolars 12.3 years, second molars 13.1 years, and third molars 19.2 years (Table 2).

#### 3.2.5 Severity

Severity was highly variable. Posterior teeth presented a higher proportion of severely compromised molars (61.9%) and premolars (70%), while anterior teeth presented a higher proportion of moderate (57.4% permanent incisors) and mild cases (37.7% primary incisors and 45.5% permanent canines) (Figure 1E–H). Only 17 teeth (10.0%) presented complete resorption with pulp compromise (Supporting Information S1: Figure 3, Table 2). In the permanent dentition, molars (OR = 27.91, 95% CI 6.22–125.18, *p* < 0.001) and premolars (OR = 64.02, 95% CI 7.69–533.19, *p* < 0.001) showed higher odds of severe or complete resorption than incisors, whereas canines did not differ significantly (OR = 1.57, 95% CI 0.23–10.93, *p* = 0.648). The ordinal sensitivity analysis agreed for molars and premolars, which remained markedly and significantly more affected than incisors, and for canines, which were not significantly different in either model, although the point estimate fell on either side of unity (Supporting Information S1: Table S2 and S2b). In the primary dentition, the five canines were all mildly affected, while of the two molars, one was severely and one completely affected; with cells of this size, no model was estimable, and the difference between tooth groups did not reach significance (Fisher's exact test, *p* = 0.066). These findings are reported descriptively.

#### 3.2.6 Histological Aspects

All five histopathological samples of resorbed teeth showed the presence of odontoclasts and/or resorption lacunae, even the trephined lateral incisor. In the four teeth extracted without previous intervention, the pulp remained vital without inflammatory cells, with a thin layer of unaffected dentine (perichamberal resorption‐resistant sheet) even in severe resorption processes (Figure 1E5). In four teeth (including the trephined lateral), a cemento‐osseous material was observed over the resorptive lacunae, two of these samples also presented bone‐like tissue not adhered to the dentine.

The three gingival tissue samples excised during gingivectomy on teeth with severe resorption (Figure 1A,F and #42 Figure 2D2) presented ectopic calcifications and nests of odontogenic epithelium (gingival hamartoma).

### 3.3 Progression of the Disease

ECR was diagnosed in 40 unerupted teeth (23.5%), 46 teeth during eruption (27%), and 84 after eruption (49.4%) (Figure 1A,B,E,G). A series of radiographs was available for 39 teeth, allowing ECR progression to be studied. Observed intervals are reported with the number of contributing teeth and patients in Supporting Information S1: Table S3; 10 of the 21 observed transitions are based on a single tooth and are described as individual cases rather than as estimates.

In primary dentition, progression could only be studied in one retained primary molar, in which the complete width of the dentine became affected within 16 months (Figure 1B).

In permanent dentition, 19 incisors were analyzed, 17 starting from the incisal edge, one from proximal surfaces, and one from both aspects simultaneously. On the incisal edge, the progression from none to moderate ECR had a median of 2.1 years (range 0.9–2.9 years, *n* = 9) and none to severe 2.7 years (*n* = 1) (Figure 1D). A different picture was observed on proximal surfaces, as ECR was not self‐limited after eruption, persisting while gingival tissue covered the area (Figure 1F). Progression from severe to complete ECR occurred in 3 years.

Four canines were analyzed, with long observed intervals: none to mild 3.5 years (*n* = 2), none to moderate 5 years (*n* = 1), and none to severe 7.9 years (*n* = 1). On average, teeth were unaffected at age 8.7 years (*n* = 3), had mild ECR at 12.3 years (*n* = 3), moderate at 13.7 years (*n* = 2), and severe at 14.7 years (*n* = 1).

In premolars, the progression was extremely variable. Observed interval from no resorption to severe had a median of 3.3 years (*n* = 4, range 2.2–6.6), however, one extremely aggressive case presented complete resorption occurring over 4 months (9.5–9.8 years, Figure 1E). In contrast, a very slow progression was observed on a premolar progressing from none to moderate over 7.9 years (6.8–14.7 years). On average, premolars were unaffected at age 10.3 (*n* = 7), had mild ECR at 12.7 (*n* = 2), moderate at 14.7 (*n* = 1), severe at 15.1 (*n* = 4), and complete at 11.3 (*n* = 2).

Based on eight molars, the observed progression interval from none to mild was 5.2 years (*n* = 1), to moderate a median of 4.5 years (*n* = 5, range 2.7–7.0), to severe 5.0 years (*n* = 5, range 2.6–7.5), and to complete 7.3 years (*n* = 2, range 5.3–9.3), with the fastest progression over 2.6 years and the slowest over 9.3 years (Supporting Information S1: Table S3).

Three particular findings were identified during this analysis: (i) Only three teeth presented resorption extending into the radicular dentine: one lateral incisor, one canine, and one molar (Figure 1G). (ii) Three teeth presented ECR starting from the mesiocervical aspect after being crowned. This was unexpected and invites monitoring with OPG even after restoration. (iii) Five teeth were retained and presented continuous slow resorption until extraction.

### 3.4 Treatment Strategies and Outcomes

In mild ECR, 47.6% of teeth received preventive/no treatment, and 52.4% were restored. Interim crowns were the most frequently used treatment (average age: primary: 1.8 years, *n* = 11, permanent: 10 years, *n* = 5), followed by composite or glass‐ionomer‐cement restorations. Gingivectomy was performed on 26.2% (Figure 2, Supporting Information S1: Figure 4 and Supporting Information S1: Table S4). Further structural loss was not recorded in the restored teeth, whereas 80% of unrestored teeth with an assessable outcome showed progressive structure loss due to attrition (Supporting Information S1: Figure 5).

In moderate ECR, 76.8% of teeth were restored (23.2% direct restorations, 46.4% interim crowns, and 7.1% ceramic crowns), 21.4% received preventive/no treatment, and 1.8% were extracted (Figure 2, Supporting Information S1: Figure 4). Further structural loss was not recorded in the restored teeth, while 57% of unrestored teeth with an assessable outcome experienced structure loss (Supporting Information S1: Figure 5).

In severe ECR, 64.9% of the teeth were restored, and no further loss of structure was recorded in them. In contrast, all the unrestored teeth with an assessable outcome experienced structure loss. 21.6% were extracted. Furthermore, 37.8% had gingivectomy and 5.4% an endodontic treatment (Figure 2).

In complete ECR, 10 of the 17 teeth could be maintained through endodontics and oral rehabilitation (58.8%). Extractions were performed in 7 teeth (41.2%), one of them (5.9%) was successfully replaced with a dental implant (Figure 2D, Supporting Information S1: Figure 4).

## 4 Discussion

ECR is an uncommon condition; however, case reports have been described in the past 80 years, and two prevalence studies have been published (Collins et al. 1999; Besa‐Witto et al. 2025). To our knowledge, this represents one of the largest reported cohorts, providing new insights into the condition, including its occurrence in primary dentition, age of detection per tooth group, progression, treatment strategies, and outcomes.

Until 2025, ECR in AI had only been reported in permanent dentition. Besa‐Witto reported ECR in primary teeth in 15 of 17 patients, in a subgroup of this cohort (Besa‐Witto et al. 2025). The present study presents 60 primary teeth with ECR from two different countries (Figure 1A,B).

The number of teeth affected per patient (average 5.7) is consistent with previous studies, such as the cohort reported by Weinmann (average 4.8, *n* = 8, range 0–8) (Weinmann et al. 1945), and a case by Korbmacher *n* = 6 (Korbmacher et al. 2007).

The classic ECR definition considers the starting point from the tip of the crown. Our study depicts that 14.6% start from other surfaces, which is clinically relevant, as resorption only occurs while the gingiva is in contact with the tooth structure, and proximal areas remain covered longer. The present findings suggest that the classical description may be too narrow. We suggest updating the definition to: “External crown resorption in Amelogenesis Imperfecta tends to start at the tip of the crown, adjacent to the oral epithelium (incisal edge or occlusal surface), however, in some cases, it can start from the proximal surface as well.”

Differential diagnosis of ECR can be challenging. Resorptive lesions are often misdiagnosed as “pre‐eruptive caries” when dentists are not aware of ECR as a possible diagnosis (Seow and Hackley 1996; Davidovich et al. 2005). Distinctive clinical signs include a lesion covered by soft tissue. At the incisal edge, it manifests as a semilunar shape. Imaging studies in this article confirm that the semilunar shape is not a primary mineralization defect, but a resorptive sequela. Evidence of well‐formed incisors with straight edges changing their shape during eruption due to ECR is found in Figures 1, 2 and 2A–D. Indeed, incisors represented the largest proportion of affected teeth in this cohort, followed by molars, canines, and premolars. This is different from the preceding literature, where a higher prevalence was found on first molars (a total of 29 cases reported), compared to incisors (only 4 cases reported). The difference can be explained as most authors used to consider semilunar edges as a shape variant, while our study has demonstrated that it reflects resorption.

Our histological findings are consistent with Weinmann's: “Even in (severe cases) the pulp chamber remains protected by a thin wall of predentine. The resorption only rarely opens the pulp chamber” (Weinmann et al. 1945), as shown in Figure 1E, where predentine and a thin layer of dentine remains unaltered. This is extremely important, together with the finding that the pulp remains vital, as it has therapeutic impact. Conservative treatment strategies with bioactive dental materials should always be considered.

Imaging studies allow pre‐eruptive diagnosis for early treatment. OPGs are considered the modality of choice for overall assessment as it enables a comprehensive evaluation of dental development and adjacent osseous structures (Okutan et al. 2025). These are indicated when clinically relevant changes can be expected, guiding therapeutic decisions. OPGs require patient cooperation, which can normally be expected from the age of 4–5 years (Kühnisch et al. 2020). In cases of insufficient visualization or limited patient cooperation, PAR may be indicated. CBCT scans aid treatment planning in complex cases, revealing lesions’ three‐dimensional aspects, such as the intact thin dentine layer in severe resorptions.

Considering radiation‐induced risks, each radiographic examination necessitates a justifying indication. Based on this cohort, we propose a provisional *radiographic screening protocol for ECR in AI associated with JEB* (Table 3) that adheres to the ALADAIP principle (As Low As Diagnostically Achievable, being indication‐oriented and Patient‐specific) (Kühnisch et al. 2020). It is based on the age of detection and progression interval presented in Table 2. Each justification is inherently linked to an expected therapeutic consequence. As the age of detection is not necessarily the age of onset, and because the data is based on irregular examination intervals, this radiographic screening protocol is a cohort‐derived proposal that will require a prospective validation. The evidence presented here is restricted to AI associated with JEB, in which hypoplastic AI is complete. Whether the timings apply to isolated and other syndromic forms of AI remains to be established.

**Table 3:** Provisional radiographic screening for external crown resorption in amelogenesis imperfecta associated with junctional epidermolysis bullosa*.

Target teeth	Age	Radiographic modality	Justifying indication for imaging study	Therapeutic strategy
Upper and lower primary incisors	~12 months if incisors are still unerupted (depending on patients’ cooperation).	PAR	Assessment of ECR to define treatment needs. ECR has been diagnosed as early as 8 months of age.	If ECR is diagnosed, a gingivectomy to remove the resorptive soft tissue should be performed to avoid further resorption. Crown tissue should be restored with a conservative approach. Interim crowns might be considered (Figure 2B). Depending on patients’ cooperation, pharmacological behavioral support might be needed.
Primary canines and molars	The available evidence from our cohort and the evidence from the available literature is not strong enough to recommend time slots for screening. Therefore, the clinical examination should focus on assessing the physiological time of eruption. If these teeth have not erupted within the normal eruption ages, considering standard deviation, PAR should be taken.		If a tooth has not erupted within expected eruption age, an imaging study should screen for ECR. ECR has been diagnosed on primary canines at the age of 1.7 years and primary molars at 4.2 years.	If ECR is diagnosed, a gingivectomy should be performed. On affected primary molars, stainless steel crowns can be considered a restorative alternative. For canines, besides adhesive restorative materials, interim crowns can be considered as an alternative (Figure 2B,C). Depending on patients’ cooperation, pharmacological behavioral support might be needed.
Upper and lower permanent incisors	~6 years if still unerupted. Follow up according to the eruption process.	PAR	Assessment of ECR to define treatment needs. ECR has been diagnosed on upper incisors from the age of 5.6 years and in lower incisors from the age of 5.2 years.	If ECR is diagnosed, a gingivectomy should be performed. Crown tissue should be restored with a conservative approach, which can be based on glass ionomer cement or resin‐based restorations up to interim crowns (Figure 2C,D). Depending on patients’ cooperation, pharmacological behavioral support might be needed.
First permanent molars	~7 years if still unerupted. Follow up according to the eruption process.	OPG	Assessment of ECR to define treatment needs. ECR has been diagnosed on first permanent molars from the age of 7.4 years.	If ECR is diagnosed, a gingivectomy should be performed. Pulp management will depend on the severity of the resorption, biomaterials to preserve pulp vitality are the first choice. Materials for crown restoration will depend on the eruption degree. In initial stages, the affected crown can be restored with direct materials such as glass ionomer cement or resin‐based materials, in further eruptive stages, the complete crown can be covered with a stainless steel crown to prevent post eruptive breakdown (Figure 2C4).
Premolars, canines, and second molars.	~10 years if unerupted. Follow up according to the eruption process.	OPG	Assessment of ECR to define treatment needs. ECR has been diagnosed on premolars from the age of 8.7 years, canines from the age of 9.3 years and second molars from the age of 11 years.	According to previously described protocols^(3)^, resorptive gingival tissue should be removed. Pulp vitality preservation should be attempted using biomaterials. Crown tissue should be restored. For canines, aesthetic demands should be considered. For premolars, stainless steel crowns should be considered.
Third molar	As time of eruption is highly variable, the need for OPG should be assessed individually.		Assessment of ECR to define treatment needs. ECR has been diagnosed on unerupted third molars from the age of 13.4 years.	Treatment strategy should consider the patient's overall treatment plan, particularly whether the third molar is planned to remain intraorally or is planned for extraction.

Abbreviations: AI, amelogenesis imperfecta; ECR, external crown resorption; JEB, junctional epidermolysis bullosa; OPG, orthopantomography; PAR, periapical radiographs.

* *Note:*

(1) Periapical radiographs have proven to be more accurate in detecting resorptive lesion, demonstrated for root resorption (Bhardwaj et al. 2017).

(2) The referenced ages correspond to the age at which ECR was first detected, rather than the age at which ECR began.

(3) Previously described treatment protocols include Luten 1958; Rankow et al. 1986; Ignelzi et al. 1990; Hall et al. 1995; Korbmacher et al. 2007.

Radiographs should carefully assess the incisal edges and proximal subgingival aspects. Special attention must be given to partially erupted teeth with hyperplastic gingival tissue next to its surface, as this can be a sign of proximal resorption.

Early ECR detection allows early intervention to avoid progressive structure loss. Treatment strategies consider removal of resorptive soft tissue invading the lesion and maintaining pulp vitality. Conservative approaches can be provided even in unerupted teeth. Gingivectomy with gentle removal of granulomatous tissue and dentine protection maintains tooth vitality and allows root development (Luten 1958; Rankow et al. 1986; Ignelzi et al. 1990; Hall et al. 1995; Korbmacher et al. 2007). Upon pulp compromise, a root canal treatment is the treatment strategy of choice (Ooya et al. 1988; Koruyucu et al. 2018). Restored teeth showed further structural loss less often than unrestored teeth, which frequently experience attrition and/or post‐eruptive fractures. Because treatment was not randomized and its allocation depended on age, eruption stage, severity, and patient cooperation, this is an association and not evidence that restoration prevents further loss. In a sensitivity analysis, the association persisted when restricted to teeth observed for at least 1 year (Supporting Information S1: Tables S5 and S6). Early restorative treatments appear clinically beneficial and should be considered whenever feasible.

Limitations of the study include available diagnostic data, depending on individual patient follow‐up frequency. No follow‐up protocols were available during the period studied, accounting for high variability in data availability; for example, the progression interval could only be estimated in 39/170 teeth, the median observation period per tooth was 2.3 years (interquartile range 0–4.5), and outcome could not be assessed in 42 teeth (24.7%). Many teeth do not have baseline imaging studies. Analysis accounted for clustering of teeth within patients, but with 30 patients, the absence of detectable clustering does not exclude it. As only patients with both JEB and ECR were eligible, the results described the distribution of affected teeth within the cohort and cannot be interpreted as the prevalence of risk. Being a cohort study, the conclusions are exploratory, requiring prospective validation.

In summary, gingivectomy, early restorative intervention, and the use of biomaterials to preserve pulp vitality are the cornerstones of maintaining a functional dentition in individuals with SAI. These strategies should be individualized within the interdisciplinary care pathway of each patient.

## Author Contributions

**Susanne Krämer:** conceptualization, design, data acquisition, analysis and interpretation, writing – draft preparation and critically revised manuscript, final approval. **Sebastián Véliz:** conceptualization, design, data acquisition, analysis and interpretation, writing – draft preparation and critically revised manuscript, final approval. **Anna‐Lena Hillebrecht:** study design, data acquisition, analysis and interpretation, writing – draft preparation and critically revised manuscript, final approval. **Ana Ortega‐Pinto:** conceptualization, histopathological data acquisition and analysis, writing – draft preparation and critically revised manuscript, final approval. **Colomba Besa:** data acquisition and interpretation, writing – critically revised manuscript, final approval. **Ignacia Fuentes:** data acquisition and interpretation, writing – critically revised manuscript, final approval. **Reinhard Schilke:** conceptualization, design, data acquisition, analysis and interpretation, writing – draft preparation and critically revised manuscript, final approval. **Jens P. Bankstahl:** data acquisition, writing – critically revised manuscript, final approval. **Elias Walter:** data acquisition and analysis, writing – critically revised manuscript, final approval. **Wiebke Semper‐Hogg:** conceptualization, data acquisition and interpretation, writing – critically revised manuscript, final approval. **Cristina Has:** conceptualization, design, data acquisition, analysis and interpretation, writing – draft preparation and critically revised manuscript, final approval. All authors gave their final approval and agreed to be accountable for all aspects of the work.

## Funding

The authors have nothing to report.

## Ethics Statement

Ethical approval was obtained at the Albert‐Ludwigs‐Universität Freiburg, Germany (EK Freiburg 21‐1171) and the Ethics Committee of the North Metropolitan Health Service, Chile (002/2023). Patients provided informed consent for the publication of images.

## Conflicts of Interest

The authors declare no conflicts of interest.

## Supporting information


Supporting File

## Data Availability

The data that support the findings of this study are available on request from the corresponding author. The data are not publicly available due to privacy or ethical restrictions.
