# Supplementary material for: Depicting External Crown Resorption in Syndromic Amelogenesis Imperfecta Associated With Junctional Epidermolysis Bullosa
Source: Clin Exp Dent Res. 2026 Oct 29;12(5):e70500. doi: 10.1002/cre2.70500 (PMC13719052; doi:10.1002/cre2.70500)
Supplement: Supplementary file 1 [file CRE2-12-e70500-s001.docx]

**SUPPLEMENTARY DATA**

**Supplementary Table S1. Co-occurrence of treatment strategies**

| **Treatment** | **No restorative treatment** | **Fluoride application** | **GIC / interim restoration** | **Direct restoration** | **Interim crown** | **Ceramic crown** | **Endodontic therapy** | **Extraction** | **Dental implant** | **Other (orthodontic traction)** | **Gingivectomy** |
| --- | --- | --- | --- | --- | --- | --- | --- | --- | --- | --- | --- |
| No restorative treatment | 10 | 0 | 0 | 0 | 0 | 0 | 0 | 0 | 0 | 0 | 1 |
| Fluoride application | 0 | 26 | 0 | 0 | 0 | 3 | 0 | 0 | 0 | 0 | 0 |
| GIC / interim restoration | 0 | 0 | 5 | 0 | 1 | 0 | 1 | 0 | 0 | 0 | 4 |
| Direct restoration | 0 | 0 | 0 | 18 | 7 | 1 | 0 | 0 | 0 | 0 | 7 |
| Interim crown | 0 | 0 | 1 | 7 | 63 | 3 | 8 | 0 | 0 | 1 | 45 |
| Ceramic crown | 0 | 3 | 0 | 1 | 3 | 12 | 2 | 0 | 0 | 1 | 3 |
| Endodontic therapy | 0 | 0 | 1 | 0 | 8 | 2 | 11 | 0 | 0 | 1 | 5 |
| Extraction | 0 | 0 | 0 | 0 | 0 | 0 | 0 | 16 | 1 | 0 | 0 |
| Dental implant | 0 | 0 | 0 | 0 | 0 | 0 | 0 | 1 | 1 | 0 | 0 |
| Other (orthodontic traction) | 0 | 0 | 0 | 0 | 1 | 1 | 1 | 0 | 0 | 1 | 1 |
| Gingivectomy | 1 | 0 | 4 | 7 | 45 | 3 | 5 | 0 | 0 | 1 | 49 |

Diagonal values indicate the total number of teeth receiving each strategy; off-diagonal values indicate teeth receiving both.

**Supplementary Table S2. Severity by tooth group, permanent dentition, binary GEE.**

| **Tooth group** | **Teeth (n)** | **Severe or complete (n)** | **Severe or complete (%)** | **OR (95% CI)** | **P value** |
| --- | --- | --- | --- | --- | --- |
| Incisors (reference) | 68 | 8 | 11.8 | 1 (reference) | Reference |
| Canines | 11 | 2 | 18.2 | 1.57 (0.23-10.93) | 0.648 |
| Premolars | 10 | 9 | 90.0 | 64.02 (7.69-533.19) | <0.001 |
| Molars | 21 | 16 | 76.2 | 27.91 (6.22-125.18) | <0.001 |

Binary generalised estimating equations (severe or complete versus mild or moderate), exchangeable working correlation, robust standard errors, clustered by patient, with permanent incisors as the reference group. This is the primary severity analysis, the proportional-odds assumption required for an ordinal model was not met (likelihood ratio test for nominal effects, p=003). Confidence intervals for premolars are very wide because only one premolar was neither severe nor complete; the proportions should be read alongside the odds ratios.

**Supplementary Table S2b. Sensitivity analysis: ordinal model with patient-level bootstrap**

| **Tooth group** | **OR (95% CI)** |
| --- | --- |
| Canines | 0.74 (0.11-10.64) |
| Premolars | 18.21 (4.68-476.91) |
| Molars | 10.31 (2.34-98.29) |

Sensitivity analysis. Ordinal (proportional-odds) model of severity by tooth group with permanent incisors as the reference group; confidence intervals from a patient-level bootstrap with 2000 resamples. Reported despite the proportional-odds assumption not being met, to show that the ranking of tooth groups does not depend on the dichotomisation used in Supplementary Table S2. 44 of 2000 resamples (2.2%) were flagged as unstable, because a tooth group had no variation in severity; their coefficients are retained, so the intervals are conservative.

**Supplementary Table S3. Observed external crown resorption progression intervals**

| **Interval type** | **Dentition** | **Tooth group** | **From** | **To** | **Teeth (n)** | **Patients (n)** | **Median (y)** | **Range (y)** | **Single tooth** |
| --- | --- | --- | --- | --- | --- | --- | --- | --- | --- |
| Consecutive | Permanent | Canine | Mild | Moderate | 1 | 1 | 4.8 | 4.8 | Yes |
| Consecutive | Permanent | Canine | Moderate | Severe | 1 | 1 | 2.9 | 2.9 | Yes |
| Consecutive | Permanent | Canine | No resorption | Mild | 2 | 1 | 3.5 | 3.5 | No |
| Consecutive | Permanent | Canine | No resorption | Moderate | 1 | 1 | 5.0 | 5.0 | Yes |
| Consecutive | Permanent | Incisor | Mild | Moderate | 2 | 2 | 1.9 | 0.3-3.4 | No |
| Consecutive | Permanent | Incisor | Moderate | Severe | 6 | 2 | 0.7 | 0.7-1.9 | No |
| Consecutive | Permanent | Incisor | No resorption | Moderate | 9 | 2 | 2.1 | 0.9-2.9 | No |
| Consecutive | Permanent | Incisor | No resorption | Severe | 1 | 1 | 2.7 | 2.7 | Yes |
| Consecutive | Permanent | Incisor | Severe | Complete | 1 | 1 | 3.0 | 3.0 | Yes |
| Consecutive | Permanent | Molar | Mild | Moderate | 1 | 1 | 1.8 | 1.8 | Yes |
| Consecutive | Permanent | Molar | Moderate | Severe | 4 | 3 | 2.5 | 1.8-4.5 | No |
| Consecutive | Permanent | Molar | No resorption | Mild | 1 | 1 | 5.2 | 5.2 | Yes |
| Consecutive | Permanent | Molar | No resorption | Moderate | 4 | 2 | 3.8 | 2.7-5.0 | No |
| Consecutive | Permanent | Molar | No resorption | Severe | 2 | 2 | 3.8 | 2.6-5.0 | No |
| Consecutive | Permanent | Molar | Severe | Complete | 2 | 2 | 1.2 | 0.5-1.8 | No |
| Consecutive | Permanent | Premolar | Mild | Moderate | 1 | 1 | 2.9 | 2.9 | Yes |
| Consecutive | Permanent | Premolar | Mild | Severe | 1 | 1 | 3.4 | 3.4 | Yes |
| Consecutive | Permanent | Premolar | No resorption | Complete | 2 | 2 | 1.3 | 0.3-2.2 | No |
| Consecutive | Permanent | Premolar | No resorption | Mild | 2 | 2 | 4.1 | 3.2-5.0 | No |
| Consecutive | Permanent | Premolar | No resorption | Severe | 3 | 2 | 3.2 | 2.2-3.4 | No |
| Consecutive | Primary | Molar | No resorption | Complete | 1 | 1 | 1.3 | 1.3 | Yes |
| Cumulative | Permanent | Canine | No resorption | Mild | 2 | 1 | 3.5 | 3.5 | No |
| Cumulative | Permanent | Canine | No resorption | Moderate | 1 | 1 | 5.0 | 5.0 | Yes |
| Cumulative | Permanent | Canine | No resorption | Severe | 1 | 1 | 7.9 | 7.9 | Yes |
| Cumulative | Permanent | Incisor | No resorption | Moderate | 9 | 2 | 2.1 | 0.9-2.9 | No |
| Cumulative | Permanent | Incisor | No resorption | Severe | 1 | 1 | 2.7 | 2.7 | Yes |
| Cumulative | Permanent | Molar | No resorption | Complete | 2 | 2 | 7.3 | 5.3-9.3 | No |
| Cumulative | Permanent | Molar | No resorption | Mild | 1 | 1 | 5.2 | 5.2 | Yes |
| Cumulative | Permanent | Molar | No resorption | Moderate | 5 | 2 | 4.5 | 2.7-7.0 | No |
| Cumulative | Permanent | Molar | No resorption | Severe | 5 | 3 | 5.0 | 2.6-7.5 | No |
| Cumulative | Permanent | Premolar | No resorption | Complete | 2 | 2 | 1.3 | 0.3-2.2 | No |
| Cumulative | Permanent | Premolar | No resorption | Mild | 2 | 2 | 4.1 | 3.2-5.0 | No |
| Cumulative | Permanent | Premolar | No resorption | Moderate | 1 | 1 | 7.9 | 7.9 | Yes |
| Cumulative | Permanent | Premolar | No resorption | Severe | 4 | 2 | 3.3 | 2.2-6.6 | No |
| Cumulative | Primary | Molar | No resorption | Complete | 1 | 1 | 1.3 | 1.3 | Yes |

Consecutive: interval between two successive observations. Cumulative: interval from the last observation without resorption to the first observation in that state; a tooth that passes through an intermediate state contributes to the cumulative interval but not to the direct consecutive one, so the two counts differ. Intervals reflect the timing of the available examinations and are therefore observed intervals, not true progression rates. Transitions based on a single tooth are individual cases, not estimates.

**Supplementary Table S4. Treatment strategies according to severity.**

| **Severity** | **Category** | **n** | **% of tooth treatments** | **% of teeth with data** | **% of all teeth** |
| --- | --- | --- | --- | --- | --- |
| Mild | Preventive / no restorative treatment | 20 | 47.6 | 51.3 | 37.0 |
| Mild | Restorative (all) | 22 | 52.4 | 53.8 | 38.9 |
| Mild | Direct restoration (incl. GIC) | 3 | 7.1 | 7.7 | 5.6 |
| Mild | Interim crown | 16 | 38.1 | 41.0 | 29.6 |
| Mild | Ceramic crown | 3 | 7.1 | 7.7 | 5.6 |
| Mild | Gingivectomy (not in denominator) | 11 | 26.2 | 28.2 | 20.4 |
| Mild | DENOMINATOR: tooth treatments (excl. gingivectomy) | 42 | 100.0 | Not applicable | Not applicable |
| Mild | Teeth with treatment data | 39 | Not applicable | 100.0 | 72.2 |
| Mild | TOTAL teeth | 54 | Not applicable | Not applicable | 100.0 |
| Moderate | Preventive / no restorative treatment | 12 | 21.4 | 24.5 | 20.7 |
| Moderate | Restorative (all) | 43 | 76.8 | 75.5 | 63.8 |
| Moderate | Direct restoration (incl. GIC) | 13 | 23.2 | 26.5 | 22.4 |
| Moderate | Interim crown | 26 | 46.4 | 53.1 | 44.8 |
| Moderate | Ceramic crown | 4 | 7.1 | 8.2 | 6.9 |
| Moderate | Extraction | 1 | 1.8 | 2.0 | 1.7 |
| Moderate | Gingivectomy (not in denominator) | 20 | 35.7 | 40.8 | 34.5 |
| Moderate | DENOMINATOR: tooth treatments (excl. gingivectomy) | 56 | 100.0 | Not applicable | Not applicable |
| Moderate | Teeth with treatment data | 49 | Not applicable | 100.0 | 84.5 |
| Moderate | TOTAL teeth | 58 | Not applicable | Not applicable | 100.0 |
| Severe | Preventive / no restorative treatment | 3 | 8.1 | 9.1 | 7.3 |
| Severe | Restorative (all) | 24 | 64.9 | 66.7 | 53.7 |
| Severe | Direct restoration (incl. GIC) | 6 | 16.2 | 18.2 | 14.6 |
| Severe | Interim crown | 14 | 37.8 | 42.4 | 34.1 |
| Severe | Ceramic crown | 4 | 10.8 | 12.1 | 9.8 |
| Severe | Endodontic therapy | 2 | 5.4 | 6.1 | 4.9 |
| Severe | Extraction | 8 | 21.6 | 24.2 | 19.5 |
| Severe | Gingivectomy (not in denominator) | 14 | 37.8 | 42.4 | 34.1 |
| Severe | DENOMINATOR: tooth treatments (excl. gingivectomy) | 37 | 100.0 | Not applicable | Not applicable |
| Severe | Teeth with treatment data | 33 | Not applicable | 100.0 | 80.5 |
| Severe | TOTAL teeth | 41 | Not applicable | Not applicable | 100.0 |
| Complete | Preventive / no restorative treatment | 1 | 3.6 | 5.9 | 5.9 |
| Complete | Restorative (all) | 9 | 32.1 | 41.2 | 41.2 |
| Complete | Direct restoration (incl. GIC) | 1 | 3.6 | 5.9 | 5.9 |
| Complete | Interim crown | 7 | 25.0 | 41.2 | 41.2 |
| Complete | Ceramic crown | 1 | 3.6 | 5.9 | 5.9 |
| Complete | Endodontic therapy | 9 | 32.1 | 52.9 | 52.9 |
| Complete | Extraction | 7 | 25.0 | 41.2 | 41.2 |
| Complete | Dental implant | 1 | 3.6 | 5.9 | 5.9 |
| Complete | Other (orthodontic traction) | 1 | 3.6 | 5.9 | 5.9 |
| Complete | Gingivectomy (not in denominator) | 4 | 14.3 | 23.5 | 23.5 |
| Complete | DENOMINATOR: tooth treatments (excl. gingivectomy) | 28 | 100.0 | Not applicable | Not applicable |
| Complete | Teeth with treatment data | 17 | Not applicable | 100.0 | 100.0 |
| Complete | TOTAL teeth | 17 | Not applicable | Not applicable | 100.0 |

Denominator: number of tooth treatments per severity group; gingivectomy is excluded from the denominator, as it is a gingival procedure, and teeth without treatment data are excluded. In the last three rows of each severity group the column n refers to teeth, not to treatments. Strategies are not mutually exclusive.

**Supplementary Table S5. Treatment strategies and follow-up**

| **Treatment** | **Teeth (n)** | **Patients (n)** | **With follow-up data (n)** | **Median follow-up (y)** | **Follow-up range (y)** |
| --- | --- | --- | --- | --- | --- |
| No restorative treatment (observation period) | 10 | 7 | 10 | 1.2 | 0.0-9.1 |
| Fluoride application (observation period) | 26 | 9 | 26 | 0.9 | 0.0-7.4 |
| GIC / interim restoration | 5 | 4 | 5 | 3.1 | 0.0-3.2 |
| Direct restoration | 18 | 5 | 17 | 4.7 | 0.5-10.2 |
| Interim crown | 63 | 10 | 63 | 3.3 | 0.0-10.2 |
| Ceramic crown | 12 | 5 | 9 | 0.6 | 0.0-10.2 |
| Endodontic therapy | 11 | 7 | 8 | 0.0 | 0.0-6.9 |
| Extraction | 16 | 8 | 0 | Not applicable | Not applicable |
| Dental implant | 1 | 1 | 0 | Not applicable | Not applicable |
| Other (orthodontic traction) | 1 | 1 | 1 | 6.9 | 6.9 |
| Gingivectomy | 49 | 8 | 49 | 3.2 | 0.0-10.2 |

Treatment strategies were not mutually exclusive; see Supplementary Table S1. No restorative treatment is compatible with gingivectomy. For preventive strategies no treatment date was recorded, and the observation period of the tooth is reported instead. Follow-up after extraction or implant placement is not applicable.

**Supplementary Table S6. Structural loss according to restoration**

| **Analysis** | **Teeth (n)** | **Loss, unrestored teeth (n/N, %)** | **Loss, restored teeth (n/N, %)** |
| --- | --- | --- | --- |
| Primary: structural loss or tooth loss (complete cases) | 128 | 30/42 (71.4%) | 0/86 (0.0%) |
| Secondary: structural loss only (extracted teeth excluded) | 112 | 14/26 (53.8%) | 0/86 (0.0%) |
| Restricted to teeth with at least 1 year of observation | 87 | 23/29 (79.3%) | 0/58 (0.0%) |
| Sensitivity: all missing assumed without loss | 170 | 30/83 (36.1%) | 0/87 (0.0%) |
| Sensitivity: all missing assumed with loss | 170 | 71/83 (85.5%) | 1/87 (1.1%) |
| Sensitivity: extreme scenario against the association | 170 | 30/83 (36.1%) | 1/87 (1.1%) |
| Sensitivity: extreme scenario favouring the association | 170 | 71/83 (85.5%) | 0/87 (0.0%) |

No odds ratio is reported. In 5 of the 7 analyses no restored tooth showed subsequent structural loss, so the maximum-likelihood estimate diverges towards zero with an unbounded confidence interval (complete separation); the remaining analyses rest on a single event among 87 restored teeth and would give an equally unstable estimate. Treatment was not randomised and allocation was influenced by age, eruption stage, severity and patient cooperation; these estimates describe an association and must not be interpreted causally.

**Supplementary Figures**

**Supplementary figure 1: Number of teeth with external crown resorption and number of diagnostic visits per patient.**

Number of teeth diagnosed with external crown resorption (ECR) and number of examinations at which ECR was diagnosed, for each of the 30 patients. Patient numbers correspond with those in table 1.

**Supplementary figure 2: Distribution of external crown resorption in Amelogenesis Imperfecta associated with Junctional Epidermolysis Bullosa per tooth in primary and permanent dentition.**

Number of teeth affected by ECR for each tooth position, in FDI notation, in the primary dentition (upper panel) and the permanent dentition (lower panel). Upper-arch teeth are shown in blue and lower-arch teeth in orange. Totals are 60 primary and 110 permanent teeth.

**Supplementary Figure 3: Severity of crown resorption in the cohort.**

Distribution of severity in the whole sample and within each tooth group, expressed as the percentage of teeth in that group. Labels inside the bars give the number of teeth in each severity category and the number below each bar gives the total for that group. Severity refers to the maximum severity recorded during follow-up.

**Supplementary Figure 4: Treatment strategies according to severity of ECR**

Percentage of tooth treatments in each severity group that corresponded to each strategy. The denominator is the number of treatments delivered to teeth in that severity group (mild 42, moderate 56, severe 37, complete 28), not the number of teeth, because a tooth could receive more than one treatment. Gingivectomy is excluded from the denominator, as it is a gingival and not dental procedure, and is expressed over that same denominator. Teeth without recorded treatment data were excluded. Strategies are not mutually exclusive.

**Supplementary Figure 5: Treatment outcomes according to severity of ECR**

Number of teeth in each outcome category, by severity and treatment group. Teeth without an assessable outcome at the last available examination are excluded (42 teeth, 24.7%). “Preventive” denotes preventive or no restorative treatment. Teeth with an assessable outcome but no recorded treatment are shown separately as “no treatment data”. Treatment was not randomised and its allocation depended on age, eruption stage, severity and patient cooperation, so these distributions describe an association and must not be interpreted causally.
